# Prognostic ability of the sTarT back screening tool for disability and pain intensity outcomes in older adults with low back pain seeking chiropractic care: a multi-national external validation study

**DOI:** 10.1186/s12998-025-00592-1

**Published:** 2025-07-30

**Authors:** Yanyan Fu, Alan D. Jenks, Sidney M. Rubinstein, Katie de Luca, Iben Axen, Bart W. Koes, Alessandro Chiarotto

**Affiliations:** 1 Department of General Practice, Erasmus MC, University Medical Center, Rotterdam, Netherlands; 2 School of Kinesiology, Faculty of Global & Community Studies, Capilano University, North Vancouver, Canada; 3 Department of Health Sciences, Faculty of Science and Amsterdam Movement Sciences research institute, Vrije Universiteit, Amsterdam, Netherlands; 4 School of Health, Medical and Applied Sciences, CQUniversity, Brisbane, Australia; 5 Institute of Environmental Medicine, Unit of Intervention and Implementation Research for Worker health, Karolinska Institutet, Stockholm, Sweden; 6 Research Unit of General Practice, Department of Public Health & Center for Muscle and Joint Health, University of Southern Denmark, Odense, Denmark

**Keywords:** Low back pain, STarT back screening tool, Older adults, Prognostic ability

## Abstract

**Background:**

Low back pain (LBP) is common among older adults, and it is a frequent reason for seeking chiropractic care. The STarT Back Screening Tool (SBT) was developed to stratify patients with LBP into low, medium, and high-risk treatment pathways, so that the treatment can be matched to each participant’s risk profile. But its prognostic performance varies across settings and populations. No studies have focused on the SBT’s utility as a stratified-care tool in older adults with LBP in a chiropractic setting. Therefore, our aim was to evaluate the ability of the SBT to predict three-, six-, and 12-month disability and pain outcomes in older adults (≥55 years) with a new episode of LBP consulting chiropractors in the Netherlands, Sweden, and Australia.

**Methods:**

This was a secondary analysis of the Back Complaints in Older Adults – Chiropractic (BACE-C) cohort. Participants visiting chiropractors with LBP completed baseline questionnaires for demographic and clinical characteristics, including the SBT. Follow-up questionnaires assessed disability (Roland Morris Disability Questionnaire (RMDQ)) and pain intensity (11-point Numerical Rating Scale (NRS)). “No improvement” on disability and pain intensity was defined as less than 30% reduction in baseline scores. We used logistic regression models to estimate discrimination metrics including the area under the receiver operating characteristic curve (AUC). Subgroup analyses were conducted by country, sex, and LBP duration; sensitivity analyses employed alternative “no improvement” definitions and linear regression on continuous outcome scores.

**Results:**

A total of 738 participants were included. The mean age of the study sample was 66.2 ± 7.5 years and 50.9% of the participants were female. The SBT showed poor discrimination for predicting no improvement in disability and pain intensity. All AUC values were below 0.60 regardless of whether SBT risk subgroups (i.e. low/medium/high) or the SBT sum score were used. Subgroup and sensitivity analyses did not meaningfully improve discrimination.

**Conclusion:**

The SBT presented limited prognostic ability to predict outcomes of disability and pain intensity in older adults with LBP in a chiropractic setting. These findings suggest insufficient evidence for the prognostic ability of the SBT risk stratification tool. Future research should explore reasons behind the limited prognostic accuracy and consider potential modifications or alternative tools.

**Supplementary Information:**

The online version contains supplementary material available at 10.1186/s12998-025-00592-1.

## Background

Low back pain (LBP) is a common symptom that imposes a significant socioeconomic burden, for example, high medical costs and work absences [1]. As the leading global cause of disability, LBP accounted for 69.0 million (95% CI: 47.9–88.9) years lived with disability (YLD) in 2020, despite a slight decrease in its proportion of all-cause YLDs worldwide compared to 1990 (8.1%, 95% CI: 6.7–9.5) [2]. LBP is a recurrent and persistent condition, associated with anxiety, depression and decreased quality of life, particularly in older populations [3]. Prevalence and years lived with disability (YLDs) due to LBP increase with age, with peak prevalence and disability rates in adults from 80 to 85 years of age [2]. Among participants seeking chiropractic care, about 16% are people over 65 years of age, with LBP-related problems being the most common reason for seeking care (56%) [4]. Information about the prognosis of older participants with LBP in the chiropractic setting can help chiropractors set appropriate treatment goals, personalize treatment plans and guide individualized clinical decisions in their management of individual participants.

The STarT Back Screening Tool (SBT) was developed to screen people with LBP to identify prognostic indicators for disability outcomes relevant to initial decision-making in primary care [5]. People are classified into three risk groups (low, medium and high) for developing persistent disability based on the SBT [5]. Multiple randomized controlled trials on adults with LBP in primary care have investigated the (cost-) effectiveness of stratified care based on the SBT and found inconsistent results. For example, stratified care was found to be effective and cost-effective in the UK [6] but not in the United States (US) and Denmark [7–9]. However, none of these trials targeted older adults, leaving us a research gap regarding the SBT’s utility in different special populations. Overall, the prognostic performance of the SBT varies by study setting, country, and age group [10–15]. Most of the studies investigating its prognostic ability have been included in recent systematic reviews, where all have found the included studies are at high-risk of bias, mainly due to shortcomings in the analysis domain [16–18]. In fact, most studies on prediction models like SBT have, so far, failed in handling missing data appropriately, in appropriate predictor selection, and in addressing modeling overfitting and optimism in model performance [18].

In the chiropractic setting, the prognostic ability of the SBT has been reported in different studies with mixed findings [19]. Some [13, 20, 21] reported that SBT risk groups have moderate associations with pain and disability outcomes while others [11, 14, 22] found poor predictive accuracy. Notably, a recent Norwegian study by Vigdal et al. [11] investigated the SBT’s performance in older adults with back pain (aged > 55) in primary care (including chiropractic) and found poor prognostic ability, although results were limited by high loss to follow-up. Despite this, no study to date has evaluated the prognostic performance of the SBT in older adults specifically in chiropractic care. To address the research gap concerning the use of the SBT in older participants with LBP in a chiropractic setting, our objective was to investigate the prognostic ability of the SBT for disability and pain intensity outcomes in individuals aged over 55 who sought chiropractic care for a new episode of LBP in the Netherlands, Sweden, and Australia.

## Methods

### Study design

Our study is a secondary analysis of data from the Back Complaints in Elders – Chiropractic (BACE-C) study, a 12-month observational, longitudinal cohort study of older adults (> 55 years) with LBP presenting for chiropractic primary care [23] in the Netherlands, Sweden and Australia. The BACE-C studies received ethical approval from their respective institutions. See Declarations for more details. The protocol of this study was registered and is accessible in Open Science Framework (https://osf.io/4n2xj/). We reported this manuscript following the transparent reporting of a multivariable prediction model for individual prognosis or diagnosis (TRIPOD) statement [24].

### Participants

Adults aged ≥ 55 years with a new episode of LBP who sought chiropractic care for their LBP were recruited from private practices in the Netherlands, Sweden, and Australia, regardless of whether they had already consulted another type of health care provider for the same episode. Participants had LBP in the region from the thoracolumbar 12th rib junction to the first sacral vertebrae, which could include pelvic pain and pain referral to the lower extremity.

Participants who were unable to complete web-based questionnaires because of language limitations or computer literacy restrictions (excluding the Australian cohort), as well as those with cognitive disorders, were excluded. In addition, participants with a suspected tumor, fracture, infection or any other potential red flag or condition considered to be a contraindication for chiropractic care were excluded.

Potentially eligible participants were briefly informed about the study procedures over the phone when they called to make an appointment or during the initial consultation with the chiropractor. Participants who agreed to participate received emails with links to informed consent and baseline questionnaires, enabling completion at home before the first appointment—or following and no later than two weeks after that initial visit. The follow-up questionnaires were sent to the participants at two weeks, six weeks, three months, six months, nine months, and at one year after the first treatment. The questionnaires were distributed by research assistants, and chiropractors had no access to the data or knowledge of baseline SBT scores. More details on the recruitment strategy are reported elsewhere [23].

### Outcomes and predictors

Outcomes variables were pain intensity measured with the 11-point numerical rating scale (NRS) [23] and disability measured with the 24-item Roland Morris Disability Questionnaire (RMDQ) [23] at three-month, six-month, and 12-month follow-ups. We categorized the RMDQ score and the NRS score as either ‘improvement’ or ‘no improvement’ in disability and pain intensity based on the following criteria: participants whose RMDQ or NRS scores show a 30% reduction from baseline (known as the minimal important change or MIC) [25, 26] were recorded as ‘Yes’ for the improvement in pain intensity or disability; otherwise, they were recorded as ‘No’.

The predictor variable was the risk subgroup (i.e. low, medium, and high-risk) to which a participant belonged, based on the SBT score measured at baseline. We aimed to test the discrimination performance using logistic regression models with two cut-offs among the three SBT risk groups. One categorical variable separated individuals in the low-risk group from those in the medium- and high-risk groups, while the other separated individuals in the low- and medium-risk groups from those in the high-risk group.

### Sample size calculation

Before data analysis, we evaluated the sample size efficiency using the method for externally validating prediction models reported by Pavlou et al. [27]. We assumed an anticipated overall outcomes proportion around 50% based on previous studies in older adults with back pain [28, 29], we expected an acceptable C-statistic of at least 0.7 [30], we set the standard error (SE) for C-statistic, calibration slope, and ‘calibration-in-the-large’ less than 0.025, 0.15 and 0.15, respectively [27], and applied the R package, *sampsizeval* [27]. This resulted in an estimated sample size of at least 428 participants.

### Missing data

We used multiple imputation with five imputed datasets and 10 iterations [31] to handle the missing values for predictors and outcomes. Complete case analyses were performed as a sensitivity analysis.

### Statistical models

We analyzed the baseline characteristics of this cohort, for example, sociodemographic variables and LBP characteristics with descriptive statistics (e.g. means and standard deviations, or medians and inter-quartile ranges). We used independent T-test for continuous variables with a normal distribution and Mann-Whitney U test for not normally distributed data, and chi-square test for categorical variables to compare the demographic variables and pain characteristics among the different SBT risk subgroups.

The prognostic ability of the SBT was evaluated using discrimination measures. For discrimination, we calculated the following metrics: sensitivity, specificity, positive and negative predictive values, positive Likelihood Ratio (LR+), negative Likelihood Ratio (LR-), and the value of the area under the receiver operating characteristic curve (AUC-ROC) where 0.5 indicates no discrimination, 0.5 to 0.7 is poor (< 0.7), 0.7 to 0.8 is acceptable (≥ 0.7 and < 0.8), 0.8 to 0.9 is excellent (≥ 0.8 and < 0.9), and above 0.9 is outstanding (≥ 0.9) [30].

We used separate logistic regression models with “no improvement in disability” and “no improvement in pain intensity” as dependent variables, and SBT risk groups as the independent variable. Given the inclusion of data from three countries (Netherlands, Sweden, and Australia) and the stratification of participants by sex and episode duration in the original SBT development study, we conducted exploratory subgroup analyses for country, sex, and LBP duration to assess whether the prognostic ability varied across different subgroups. To investigate the prognostic ability of the SBT risk groups more comprehensively, we conducted a sensitivity analysis for disability using the original study’s definition of “no improvement”, defined as RMDQ scores ≥ 7 at follow-ups [5], and another sensitivity analysis applying the SBT sum score as predictor in separate logistic models. Since dichotomizing continuous outcome variables (e.g., NRS and RMDQ scores) can result in information loss and reduced statistical power [32], we also performed a sensitivity analysis with linear models using continuous outcome variables to maintain consistency with the primary analysis. Additionally, we reported the R² value to explain the variance in our linear regression models. The analyses were performed with SPSS version 29.0.1.0.

## Results

### Participants and baseline characteristics

This analysis included 738 participants at baseline from the multi-national BACE-C cohort, comprising 217 (29%) participants from the Netherlands, 301 participants (41%) from Sweden, and 220 (30%) from Australia. The mean age of the study sample was 66.2 (SD 7.5) years and approximately half of the participants were female (Table 1). In total, 89.6% of participants had experienced LBP before, and 43.2% met the criteria for chronic LBP (lasting ≥ three months). The mean NRS score (pain intensity) over the prior week was 5.9 (SD 2.3), and the mean RMDQ score (disability) was 8.8 (SD 5.8). Most baseline characteristics such as education, marital status, comorbidities LBP chronicity or LBP history, varied among the three countries, while a few demographic and clinical profiles (e.g., age, pain intensity) were similar (Table 1).

**Table 1:** Characteristics of the BACE-C patients at baseline (*n* = 738)

	Mean ± SD or *n* (%)				Total Missing (*n*%)
	Total population	Netherlands (*n* = 217) *	Sweden (*n* = 301) *	Australia (*n* = 220) *	
**Patient characteristics**					
Age (years)	66.2 ± 7.5	66.3 ± 7.8	65.3 ± 6.8	67.4 ± 7.8	6 (1.1)
Sex (female)	376 (50.9)	100 (46.1)	164 (54.5)	112 (50.9)	8 (1.1)
Body mass index	26.9 ± 4.8	26.3 ± 4.3	26.7 ± 4.6	27.9 ± 5.3	12 (1.6)
Educational level					
Low	183 (24.8)	85 (39.2)	30 (10.0)	68 (30.9)	5 (0.7)
Middle	223 (30.2)	49 (22.6)	146 (48.5)	28 (12.7)	
High	279 (37.8)	82 (37.8)	125 (41.5)	72 (32.7)	
Vocational Education (only for Australia)	48 (6.5)	Not applicable	Not applicable	48 (21.8)	
Marital status					
Married	307 (41.6)	30 (13.8)	222 (73.8)	55 (25.0)	5 (0.7)
Living together	42 (5.7)	7 (3.2)	15 (5.0)	20 (9.1)	
Single	374 (50.7)	179 (82.5)	63 (20.9)	132 (60.0)	
Other (only for Australia)	10 (1.4)	Not applicable	Not applicable	10 (4.5)	
Job (Employed)	237 (32.1)	72 (33.2)	128 (42.5)	37 (16.8)	9 (1.2)
Heavy drinking risk (high and severe risk)	Not applicable	122 (56.2)	Not applicable	45 (20.5)	51 (23.5) for Netherlands, 6 (2.7) for Australia
Smoke (Yes)	Not applicable	25 (11.5)	Not applicable	126 (57.3)	24 (11.1) for Netherlands, 4 (1.8) for Australia
**LBP characteristics**					
LBP^a^ history (Yes)	596 (80.8)	176 (81.1)	234 (77.7)	186 (84.5)	5 (0.7)
LBP chronicity (> 3 months)	319 (43.2)	107 (49.3)	129 (42.9)	83 (37.7)	36 (4.9)
Radiating pain (Yes)	312 (42.3)	129 (59.4)	115 (38.2)	68 (30.9)	14 (1.9)
LBP intensity last week (NRS^b^)	5.9 ± 2.3	5.9 ± 2.2	5.9 ± 2.3	5.7 ± 2.4	3 (0.4)
Medication^f^ (Yes)	266 (36.0)	110 (50.7)	116 (38.5)	40 (18.2)	145 (19.6)
Comorbidities (SCQ^c^ sum score)	2.5 ± 3.0	4.9 ± 4.3	1.7 ± 2.0	1.6 ± 1.4	35 (4.7)
STarT back tool					
Low risk	450 (61.0)	130 (59.9)	190 (63.1)	130 (59.1)	42 (5.7)
Medium risk	201 (27.2)	67 (30.9)	75 (24.9)	59 (26.8)	
High risk	45 (6.1)	18 (8.3)	10 (3.3)	17 (7.7)	
Disability (RMDQ^d^)	8.8 ± 5.8	9.5 ± 5.8	9.8 ± 5.7	6.7 ± 5.4	32 (4.3)
**Psychological characteristics**					
EQ-5D-5 L (VAS^e^)	Not applicable	69.6 ± 16.8	Not applicable	Not applicable	2 (0.9) for Netherlands
EQ-5D-5 L (index)	Not applicable	0.7 ± 0.2			3 (1.4) for Netherlands
EQ-5D-3 L (VAS)	Not applicable	Not applicable	71.5 ± 19.0	73.1 ± 15.9	0 (0) for Sweden 14 (6.4) for Australia
EQ-5D-3 L (index)	Not applicable		0.9 ± 0.1	0.7 ± 0.5	10 (3.3) for Sweden 12 (5.5) for Australia

a: low back pain; b: the Numeric Rating Scale for pain; c: Self-Administered Comorbidities Questionnaire; d: 24-item Roland Morris Disability Questionnaire; e: Visual analogue scale; index: Health state index score of Eq. 5D5L and Eq. 5D3L; f: any prescription medications for patients with LBP; * Total number of patients in each country without accounting missing values

In the total population, 61.0% of participants were classified as low risk of poor disability outcome, 27.2% as medium risk, and 6.1% as high risk based on the SBT (Table 1). When we compared the demographic and clinical characteristics among the different SBT risk subgroups (Table 2), we found that mean age varied significantly (*p* < 0.001), with participants in the high-risk group being relatively older (68.4 (SD 9.0) years) than those in the low or medium risk groups. The high-risk group had the lowest mean BMI (24.9 (SD 5.0)) as compared to the low and medium risk groups (*p* < 0.001). Pain intensity and disability were both statistically higher (*p* < 0.001) in the medium and high-risk group compared with the low-risk group (Table 2). We also compared the baseline characteristics across SBT risk subgroups in different countries, as reported in supplementary Table 1s in the appendices.

**Table 2:** Baseline characteristics comparison among start back tool risk subgroups (*N* = 738)

	SBT risk group (mean ± SD or *n* (%))			Missing(*n*%) ^#^
	Low (*n* = 450) *	Medium(*n* = 201) *	High (*n* = 45) *	
**Total population**				
** Patient characteristics**				
Age (years)	66.4 ± 7.2	64.5 ± 7.2	68.2 ± 8.3	46 (6.2)
Sex (female)	219 (48.7)	112 (55.7)	23 (51.1)	49 (6.6)
Body mass index	26.5 ± 4.5	28.0 ± 5.0	27.0 ± 5.0	53 (7.2)
Job (Employed)	134 (29.8)	79 (39.3)	9 (20.0)	48 (6.5)
** LBP**^**a**^ **characteristics**				
LBP history (Yes)	357 (79.3)	169 (84.1)	38 (84.4)	44 (6.0)
LBP chronicity (> 3 months)	202 (44.9)	87 (43.3)	18 (40.0)	73 (9.9)
Radiating pain (Yes)	182 (40.4)	91 (45.3)	24(53.3)	47 (6.4)
LBP intensity last week (NRS^b^)	5.3 ± 2.1	6.9 ± 1.9	7.5 ± 2.1	43 (5.8)
Medication^f^ (Yes)	137 (30.4)	99 (49.3)	21 (46.7)	166 (22.5)
Comorbidities (SCQ^c^ sum score)	2.4 ± 2.8	3.1 ± 3.4	3.3 ± 4.8	72 (9.8)
Disability (RMDQ^d^)	6.4 ± 4.7	12.5 ± 4.6	15.9 ± 5.0	62 (8.4)

a: low back pain; b: the Numeric Rating Scale for pain; c: Self-Administered Comorbidities Questionnaire; d: 24-item Roland Morris Disability Questionnaire; f: any prescription medications for patients with LBP; * Total number of patients in each risk group accounting missing values; # Number and percentage of missing value for all risk group

### Outcome variables

**Fig. 1:**
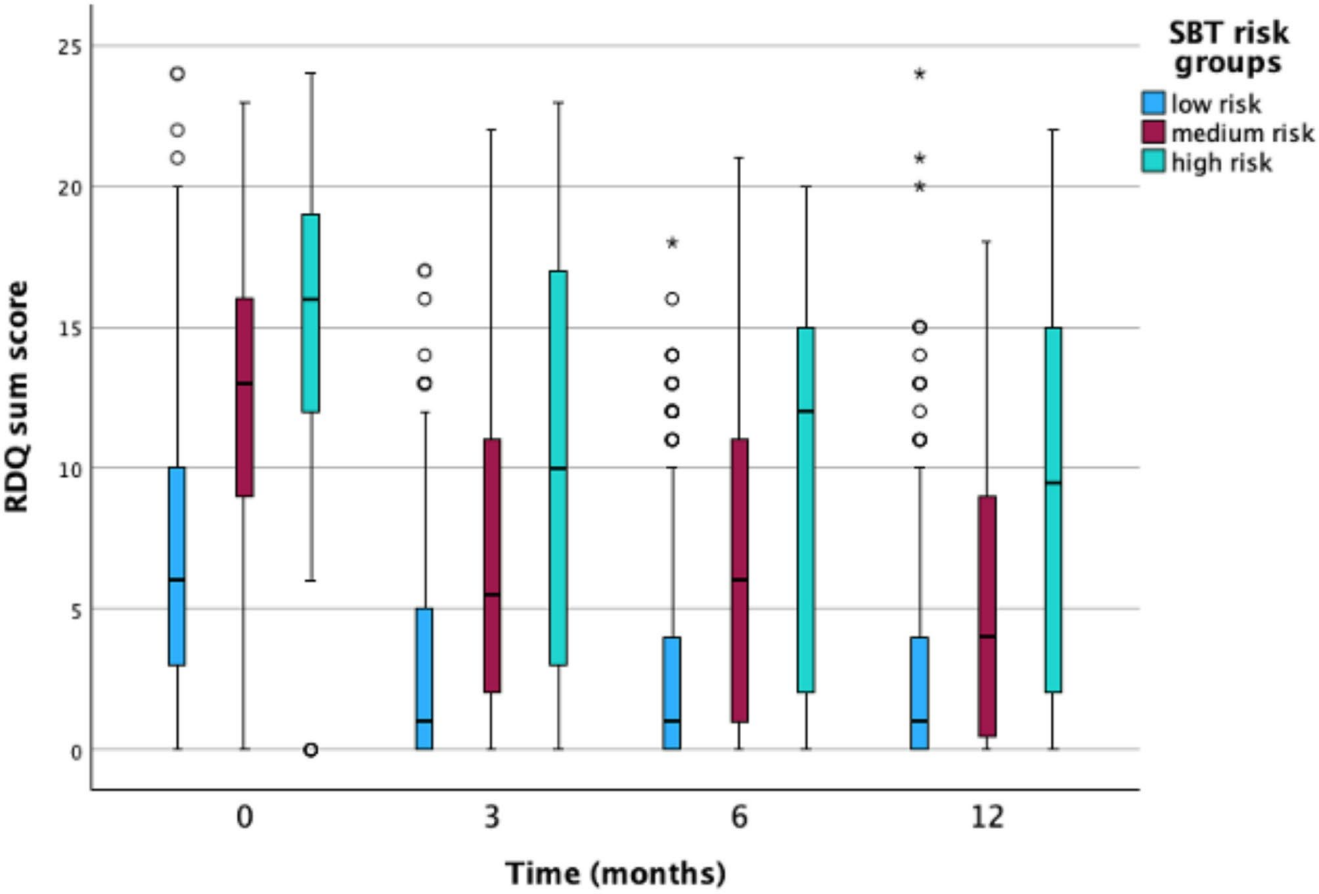
Box plots for disability outcomes stratified by SBT risk groups

**Fig. 2:**
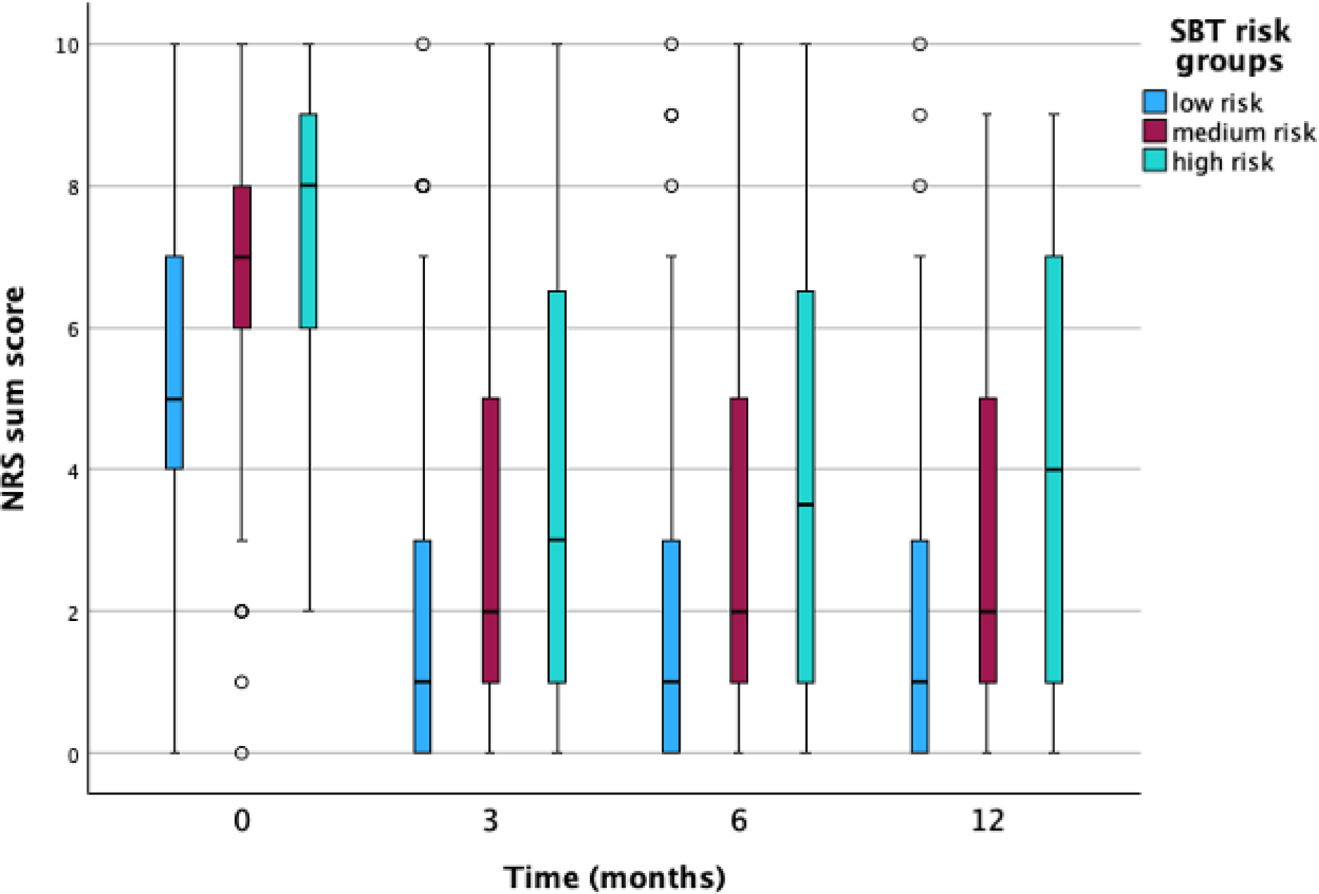
Box plots for pain intensity outcomes stratified by SBT risk groups

At three months, the mean RMDQ was 4.3 (SD 5.0), with 170 participants (23.0%) missing RMDQ data; the three months mean NRS was 2.3 (SD 2.3), with 148 participants (20.1%) missing NRS data. At six months, RMDQ scores were similar with RMDQ score at three months (mean 4.2, SD 5.1), but there was a higher proportion of missing data (231 participants, 31.3%). The mean NRS score at six months was 2.3 (SD 2.3), with 214 participants (29.0%) missing. By the 12-month follow-up, the mean RMDQ score had decreased slightly to 3.6 (SD 4.9), whereas the mean NRS had increased to 3.8 (SD 4.9). Missing data at 12 months were 235 participants (31.8%) for RMDQ and 221 (29.9%) for NRS, respectively. To visualize the distribution, variance, and potential outliers in disability and pain intensity scores, we present the box plots for outcome variables stratified by SBT risk groups in Figs. 1 and 2.

### Prognostic ability of the SBT

**Fig. 3:**
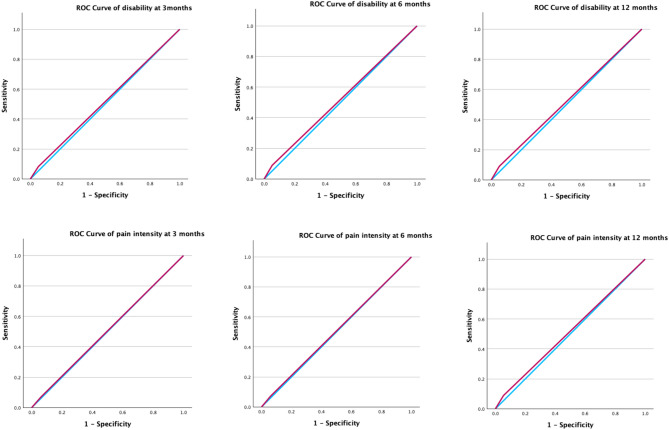
The ROC curves of the Low vs Medium and High cut-off values on disability and pain intensity outcomes

The AUC values for distinguishing participants without improvement in pain intensity and disability were low for both the cut-off value between low and medium risk group, and the cut-off value between medium and high-risk group. The AUC values ranged from 0.505 to 0.549 indicating a poor discrimination for both disability and pain intensity outcomes at three, six, and 12 months (Table 3). When comparing individuals in the low-risk group versus those in the medium or high-risk group, the sensitivity for identifying “no improvement” in disability or pain intensity was generally between 0.374 and 0.424, whereas specificity ranged from 0.651 to 0.675 (Table 3). For the other cut-off value (low and medium risk group vs. high-risk group) showed high specificity from 0.945 to 0.949 but low sensitivity from 0.073 to 0.096 (Table 3). Subgroup analyses (Supplementary Table 2s to Table 4s in appendixes) for country (Netherlands, Sweden, Australia), sex, and LBP chronicity showed consistent results with the primary analysis. We present the ROC curve plots of the Low vs. Medium and High cut-off values for disability and pain intensity at 3, 6, and 12 months in Fig. 3 to visualize the main AUC results. Since the analysis was based on five imputed datasets, five ROC curves were generated for each outcome. Therefore, only the first ROC curve was shown. Additional ROC curves for disability and pain outcomes (Low vs. Medium/High cut-offs) are presented in Fig. S1s of the supplementary file.

**Table 3:** Discrimination of the start back risk subgroups for predicting NO improvement in disability and pain intensity (*N* = 738)

	AUC (95%CI)	Sensitivity	Specificity	PPV^h^	NPV^i^	LR + ^j^	LR-^k^
**SBT**^**g**^ **risk group cut-off: Low (n = 450) VS Medium and High (n = 246)**							
Disability improvement at 3 months	0.534 (0.479, 0.590)	0.402	0.667	0.380	0.687	1.206	0.897
Disability improvement at 6 months	0.549 (0.499, 0.599)	0.424	0.674	0.365	0.726	1.299	0.855
Disability improvement at 12 months	0.537 (0.487, 0.588)	0.409	0.665	0.329	0.738	1.223	0.888
Pain improvement at 3 months	0.512 (0.459, 0.565)	0.374	0.650	0.260	0.759	1.069	0.963
Pain improvement at 6 months	0.544 (0.491, 0.597)	0.423	0.655	0.291	0.780	1.264	0.867
Pain improvement at 12 months	0.537 (0.480, 0.593)	0.412	0.664	0.290	0.772	1.226	0.886
**SBT risk group cut-off: Low and Medium (n = 651) VS High (n = 45)**							
Disability improvement at 3 months	0.518 (0.469, 0.566)	0.089	0.947	0.459	0.672	1.665	0.963
Disability improvement at 6 months	0.520 (0.473, 0.567)	0.093	0.947	0.437	0.702	1.751	0.958
Disability improvement at 12 months	0.522 (0.473, 0.571)	0.097	0.947	0.424	0.724	1.837	0.954
Pain improvement at 3 months	0.505 (0.456, 0.555)	0.073	0.938	0.280	0.754	1.177	0.988
Pain improvement at 6 months	0.508 (0.457, 0.558)	0.076	0.939	0.288	0.758	1.244	0.984
Pain improvement at 12 months	0.517 (0.466, 0.567)	0.090	0.943	0.346	0.756	1.581	0.965

g: the STarT Back Tool; h: positive predictive values; i: negative predictive values; j: Positive Likelihood Ratio; k: Negative Likelihood Ratio

**Table 4:** Discrimination of the start back sum score for predicting *NO* improvement in disability and pain intensity (*N* = 738)

	AUC (95%CI)	Sensitivity	Specificity	PPV^h^	NPV^i^	LR + ^j^	LR-^k^
Disability at 3 months	0.525 (0.462–0.588)	0.399	0.670	0.380	0.687	1.206	0.898
Disability at 6 months	0.546 (0.484, 0.608)	0.423	0.677	0.366	0.726	1.307	0.853
Disability at 12 months	0.540 (0.490, 0.590)	0.406	0.667	0.328	0.737	1.220	0.890
Pain at 3 months	0.500 (0.442, 0.558)	0.368	0.651	0.258	0.758	1.055	0.971
Pain at 6 months	0.515 (0.455, 0.575)	0.422	0.669	0.292	0.781	1.273	0.865
Pain at 12 months	0.515 (0.452, 0.577)	0.411	0.666	0.291	0.772	1.231	0.884

h: positive predictive values; i: negative predictive values; j: Positive Likelihood Ratio; k: Negative Likelihood Ratio

Table 4 presents similar analyses using the SBT sum score as a continuous predictor in separate logistic models. The results were broadly consistent, with AUC values from 0.511 to 0.545 for disability and pain outcomes. The sensitivity for identifying “no improvement” in disability or pain intensity ranged from 0.382 to 0.418, and the specificity ranged from 0.656 to 0.675. Subgroup analyses of SBT sum score (Supplementary Table 13 s to Table 15 s in appendixes) for country (Netherlands, Sweden, Australia), sex, and LBP chronicity showed consistent results with the primary analysis.

Sensitivity analyses (in appendixes) which applied alternative “no improvement” definitions (i.e., RMDQ score at follow-up time ≥ 7) (Supplementary present in Table 9 s to Table 12 s) or used linear regression on the continuous RMDQ and NRS scores (Supplementary present in Table 5 s to Table 8 s), did not produce acceptable AUC values either (> 0.7) or displayed low R²values (R²values < 0.2 in all linear models).

Our original protocol included evaluating model calibration. However, the logistic regression models (using only the SBT risk groups as predictor) produced nearly “perfect” calibration intercepts (0) and slopes (1) that were not clinically meaningful. This may occur because when the predictor set is very limited (only one predictor in our models) or when subgroup sizes are small (e.g., only 6.5% of participants fall into the high-risk SBT group in our study), the predicted probabilities tend to align closely with the observed events [33–36]. As a result, we omitted the calibration estimates to avoid presenting misleading conclusions.

## Discussion

This study assessed the prognostic ability of the SBT on disability and pain intensity at three-, six-, and 12-month follow-ups for older adults with low back pain seeking chiropractic care. The SBT showed limited discrimination ability with AUC values under 0.7, irrespective of whether the tool was used in its cut-off values of low/medium/high risk groups or continuous sum score. Moreover, subgroup analyses stratified by country (Netherlands, Sweden, Australia), sex, and LBP chronicity confirmed the primary findings, showing no notable improvements in prognostic performance in any subgroup. Sensitivity analyses that used the same “no improvement” definitions as in the original SBT development study (RMDQ score > 7/24) produced somewhat better AUC values than other models. However, the AUC values remained below 0.7, which still represents poor discrimination.

The specificity and sensitivity for cut-off between Low vs. Medium/High was relatively balanced, while cut-off between Low/Medium vs. High produced very high specificity but very poor sensitivity, which is caused by the imbalanced distribution of participants across three SBT risk group (64.7% for low, 28.8% for medium and 6.5% for the high-risk group). This cut-off lumped low and medium SBT risk groups as “negative” and defined only the high-risk group as “positive,” so the small proportion of high-risk participants increased specificity while missing many who developed persistent disability (very low sensitivity) [33].

Vigdal et al. study focusing on older adults [11] reported that the SBT risk groups were not accurate in predicting poor LBP outcomes, specifically persistent disability, which aligns with our overall findings. Unlike our study, Vigdal et al. used the same definition of disability outcome (RMDQ score > 7/24) as the original development study of the SBT. The discrimination ability of the model was poor in both studies, with an AUC below 0.7. However, our study showed a lower AUC value than Vigdal’s study. Our AUC values for persistent disability were all below 0.6, while Vigdal et al. reported AUCs of 0.65 (0.59–0.71), 0.67 (0.60–0.73), and 0.65 (0.58–0.71) at 3, 6, and 12 months, respectively. One possible interpretation for that is the difference of composition of the study population, Vigdal’s study population included participants from general practitioners (GPs), physical therapists (PTs) and chiropractors, while our study population was only recruited from chiropractors. In the sensitivity analysis, when using the same definition of poor outcomes, the AUC values at the cut-off of low vs. medium/high-risk groups were like those of Vigdal’s study, ranging from 0.638 to 0.698. The AUC is a measure of how well a classifier (such as the SBT) can discriminate between two groups. Changes in the outcome definition can redraw the boundaries of group classifications, which in turn can affect the model’s performance metrics, including the AUC [33]. Our outcome definition accounts for each participant’s baseline value instead of an absolute RMDQ score. However, it may also reduce the proportion of older adults classified with persistent disability, thereby lowering the AUC—especially for participants with RMDQ scores higher than 7 at both baseline and follow-up time. For instance, according to Hill et al., with a baseline RMDQ of 10 and a 6‑month RMDQ of 7 would be classed as persistent disability group. In contrast, based on our definition, this participant would be classified as “no persistent disability” because of a 30% reduction of RMDQ score from baseline.

Compared with our findings in older adults in the chiropractic setting (AUC values below 0.60), the STarT Back Screening Tool has shown poor to acceptable discrimination ability (AUC < 0.7 reported by Kongsted et al. [22] and Vigdal et al. [11] and AUC = 0.76 report by Szita et al. [37]) across various settings (such as Danish physiotherapy [12], US primary care [15], and physical therapy [10, 37]) in predicting poor low back pain outcomes. However, none of these previous validation studies has reported AUCs that reach the “excellent” (≥ 0.80) or “outstanding” (≥ 0.90) thresholds. This suggests that the SBT’s prognostic ability may be limited in older adults seeking chiropractic care highlighting the importance of clinical setting for applying the SBT in clinical practice.

Differences in participant demographics (e.g., older vs. younger adults), clinical environments (e.g., chiropractic vs. primary care), or the different definition of “poor outcome” appear to drive the variability in SBT accuracy across studies. These can reclassify participants, thereby altering sensitivity, specificity, and the overall AUC. Moreover, older adults commonly present with comorbidities and complex health conditions [38–40], unlike the younger and healthier population in primary care settings included in the original development study [5]. The difference may weaken the predictive ability of a tool like SBT, which was originally developed for more general primary care populations.

While the SBT prognostic performance is limited in older adults with LBP, it may be worthwhile to consider other prognostic tools or models in this participant population. For example, in a systematic review with meta-analysis, Karran and colleagues found that the Orebro Musculoskeletal Pain Screening Questionnaire (OMPSQ) displays higher predictive accuracy, when compared to the SBT, in adult participants with recent onset LBP [41]. Moreover, always in older adults with LBP, the BACE-D (Back Complaints in the Elders-Dutch cohort study) prognostic models displayed acceptable to excellent performance at predicting outcomes like pain intensity and disability at both development and internal validation [29], and external validation [42]. The BACE models included various predictors which, in contrast with the predictors included in the SBT, were non-modifiable, such as age, LBP duration, recent LBP history, and participants’ expectation for recovery. This underscores the importance of including also non-modifiable prognostic factors to have a more accurate estimation of the likely prognosis of older adults with LBP in primary care.

Although SBT presented poor discrimination in our study, a RCT in a primary care setting in the UK showed that stratified care based on SBT reduced disability and was cost-effective, whereas a similar US trial found no benefit [6, 7]. A narrative review suggests these conflicting results might be caused by implementation barriers across varied healthcare systems rather than the flaws in SBT’s risk-prediction accuracy [43].

Our research shows that the prognostic accuracy of SBT is poor in chiropractic settings, indicating that chiropractors should not rely on the SBT risk groups to guide the treatment decisions for older patients with LBP. In future research, we should investigate why the prognostic accuracy of SBT is reduced in older adults seeking chiropractic care. Possible reasons may include differences in participant characteristics (e.g., comorbidities, pain duration, psychological factors) or variations in chiropractic care practices compared with primary care. The variation in chiropractic practice patterns across countries and providers might reduce the generalizability and predictive ability of the SBT in these specific settings [13]. Studying these reasons may lead to modified versions of SBT, new prognostic tools, or a further understanding of why the SBT performs inconsistently in predicting LBP outcomes in this population.

### Strengths and limitations

A strength of this study is its large, international design, enhancing the generalizability of our findings to other chiropractic settings. However, there are several limitations in our study. First, our study was a secondary analysis of the BACE-C cohort, which was not originally designed to validate the SBT specifically in a population of older chiropractic participants. Second, there was a substantial amount of missing data at follow-ups (ranging from 23.0 to 29.9%). Even with multiple imputation, it may still introduce additional variability, and assumption that data were missing at random may not hold true in all cases.

## Conclusion

In this external validation study of older adults seeking chiropractic care for low back pain, the SBT demonstrated limited ability to discriminate those having no improvement from those having improvement in disability and pain intensity outcomes at three-, six-, and 12-month follow-ups. Whether analyzed using the original risk groups cut-offs or treated as a continuous sum score, the SBT consistently provided low sensitivity and specificity values, as well as AUCs below 0.7. These results suggest that there is no strong argument for using the SBT to predict long-term pain and disability outcomes in older adults with LBP in chiropractic settings. Future research should focus on the reasons behind the limited prognostic performance observed in this population, which may lead to the modification of the SBT or the development of a new tailored instrument.

## Supplementary Information

Supplementary Material 1

Supplementary Material 2

Supplementary Material 3

## Data Availability

No datasets were generated or analysed during the current study.

## Abbreviations

AUC: Area Under the Receiver Operating Characteristic Curve
BACE-C: Back Complaints in Elders–Chiropractic
BACE-D: Back Complaints in Elders–Dutch
BMI: Body Mass Index
CI: Confidence Interval
GBD: Global Burden of Disease
LBP: Low Back Pain
LR+: Positive Likelihood Ratio
LR-: Negative Likelihood Ratio
MIC: Minimal Important Change
NRS: Numerical Rating Scale
R²: Coefficient of Determination
RMDQ: Roland–Morris Disability Questionnaire
SBT: STarT Back Screening Tool
SD: Standard Deviation
SE: Standard Error
SPSS: Statistical Package for the Social Sciences
TRIPOD: Transparent Reporting of a Multivariable Prediction Model for Individual Prognosis or Diagnosis
UK: United Kingdom
YLDs: Years Lived with Disability
