# Supplementary material for: Prognostic ability of the sTarT back screening tool for disability and pain intensity outcomes in older adults with low back pain seeking chiropractic care: a multi-national external validation study
Source: Chiropr Man Therap. 2025 Jul 30;33:30. doi: 10.1186/s12998-025-00592-1 (PMC12312513; doi:10.1186/s12998-025-00592-1)

**Figure 1s. The ROC curves of the Low and Medium vs High cut-off values on disability and pain intensity outcomes**


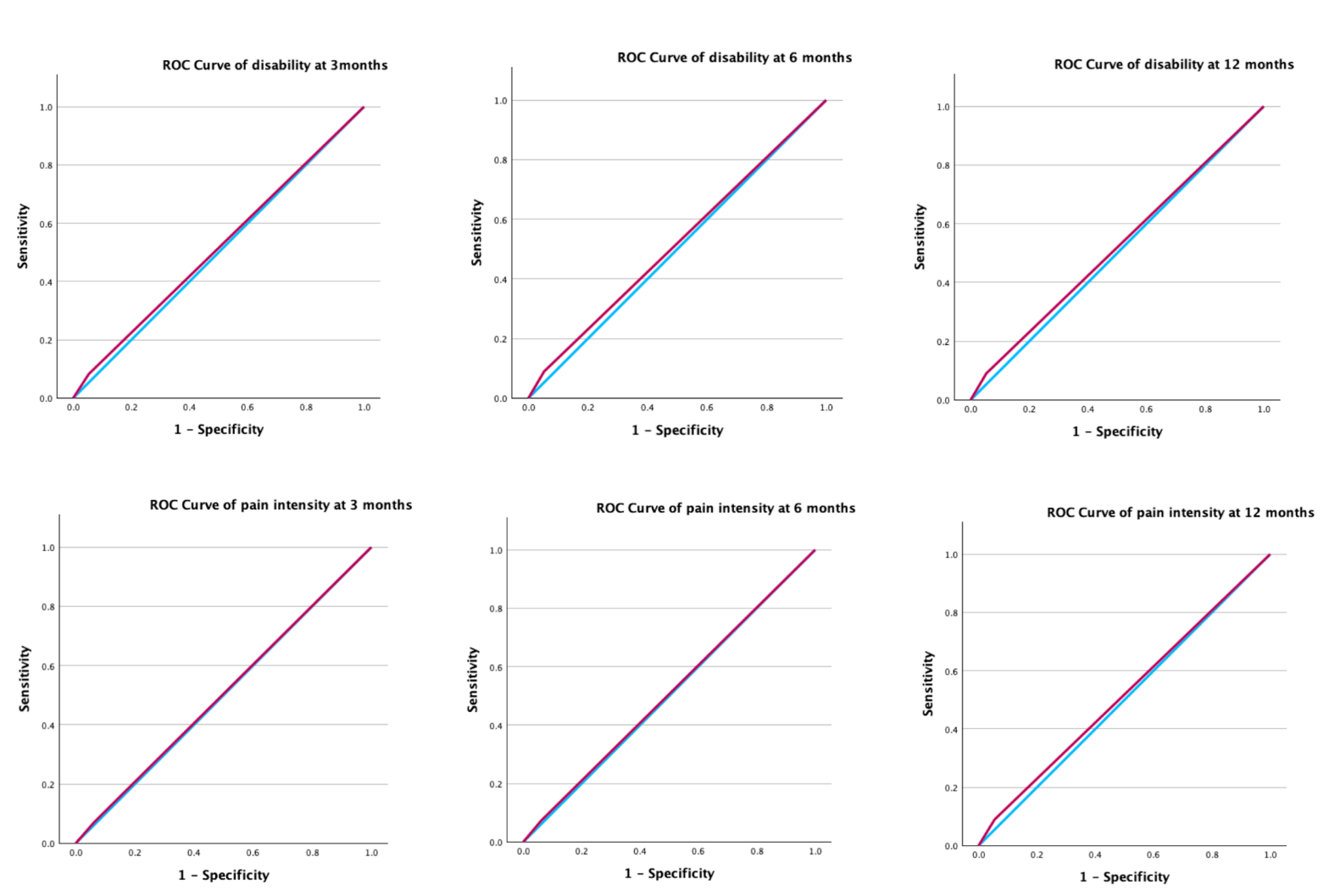

Supplement: Supplementary file 1 — Supplementary Material 1 [file 12998_2025_592_MOESM1_ESM.docx]
