# Supplementary material for: Prognostic ability of the sTarT back screening tool for disability and pain intensity outcomes in older adults with low back pain seeking chiropractic care: a multi-national external validation study
Source: Chiropr Man Therap. 2025 Jul 30;33:30. doi: 10.1186/s12998-025-00592-1 (PMC12312513; doi:10.1186/s12998-025-00592-1)
Supplement: Supplementary file 2 — Supplementary Material 2 [file 12998_2025_592_MOESM2_ESM.pdf]

Table

Table 1| Checklist of items to include when reporting a study developing or validating a multivariable prediction model for diagnosis or prognosis*

Section/topic	Item	Development or validation?	Checklist item	Page
**Title and abstract**				
Title	1	D;V	Identify the study as developing and/or validating a multivariable prediction model, the target population, and the outcome to be predicted	
Abstract	2	D;V	Provide a summary of objectives, study design, setting, participants, sample size, predictors, outcome, statistical analysis, results, and conclusions	
**Introduction**				
Background and objectives	3a	D;V	Explain the medical context (including whether diagnostic or prognostic) and rationale for developing or validating the multivariable prediction model, including references to existing models	
	3b	D;V	Specify the objectives, including whether the study describes the development or validation of the model, or both	
**Methods**				
Source of data	4a	D;V	Describe the study design or source of data (for example, randomised trial, cohort, or registry data), separately for the development and validation data sets, if applicable	
	4b	D;V	Specify the key study dates, including start of accrual; end of accrual; and, if applicable, end of follow-up	
Participants	5a	D;V	Specify key elements of the study setting (for example, primary care, secondary care, general population) including number and location of centres	
	5b	D;V	Describe eligibility criteria for participants	
	5c	D;V	Give details of treatments received, if relevant	
Outcome	6a	D;V	Clearly define the outcome that is predicted by the prediction model, including how and when assessed	
	6b	D;V	Report any actions to blind assessment of the outcome to be predicted	
Predictors	7a	D;V	Clearly define all predictors used in developing the multivariable prediction model, including how and when they were measured	
	7b	D;V	Report any actions to blind assessment of predictors for the outcome and other predictors	
Sample size	8	D;V	Explain how the study size was arrived at.	
Missing data	9	D;V	Describe how missing data were handled (for example, complete-case analysis, single imputation, multiple imputation) with details of any imputation method	
Statistical analysis methods	10a	D	Describe how predictors were handled in the analyses	
	10b	D	Specify type of model, all model-building procedures (including any predictor selection), and method for internal validation	
	10c	V	For validation, describe how the predictions were calculated	
	10d	D;V	Specify all measures used to assess model performance and, if relevant, to compare multiple models	
	10e	V	Describe any model updating (for example, recalibration) arising from the validation, if done	
Risk groups	11	D;V	Provide details on how risk groups were created, if done	
Development *v* validation	12	V	For validation, identify any differences from the development data in setting, eligibility criteria, outcome, and predictors	
**Results**				
Participants	13a	D;V	Describe the flow of participants through the study, including the number of participants with and without the outcome and, if applicable, a summary of the follow-up time. A diagram may be helpful	
	13b	D;V	Describe the characteristics of the participants (basic demographics, clinical features, available predictors), including the number of participants with missing data for predictors and outcome	
	13c	V	For validation, show a comparison with the development data of the distribution of important variables (demographics, predictors and outcome).	
Model development	14a	D	Specify the number of participants and outcome events in each analysis	

BMJ: first published as 10.1136/bmj.g7594 on 7 January 2015. Downloaded from https://www.bmj.com/ on 3 March 2025 by guest. Protected by copyright, including for uses related to text and data mining, AI training, and similar technologies.

For personal use only: See rights and reprints http://www.bmj.com/permissions Subscribe: http://www.bmj.com/subscribe

(continued)

Section/topic	Item	Development or validation?	Checklist item	Page
	14b	D	If done, report the unadjusted association between each candidate predictor and outcome	
Model specification	15a	D	Present the full prediction model to allow predictions for individuals (that is, all regression coefficients, and model intercept or baseline survival at a given time point)	
	15b	D	Explain how to use the prediction model	
Model performance	16	D;V	Report performance measures (with CIs) for the prediction model	
Model updating	17	V	If done, report the results from any model updating (that is, model specification, model performance)	
**Discussion**				
Limitations	18	D;V	Discuss any limitations of the study (such as nonrepresentative sample, few events per predictor, missing data)	
Interpretation	19a	V	For validation, discuss the results with reference to performance in the development data, and any other validation data	
	19b	D;V	Give an overall interpretation of the results, considering objectives, limitations, results from similar studies, and other relevant evidence	
Implications	20	D;V	Discuss the potential clinical use of the model and implications for future research	
**Other information**				
Supplementary information	21	D;V	Provide information about the availability of supplementary resources, such as study protocol, Web calculator, and data sets	
Funding	22	D;V	Give the source of funding and the role of the funders for the present study	

*Items relevant only to the development of a prediction model are denoted by *D*, items relating solely to a validation of a prediction model are denoted by *V*, and items relating to both are denoted *D;V*. We recommend using the TRIPOD Checklist in conjunction with the TRIPOD explanation and elaboration document.

BMJ: first published as 10.1136/bmj.g7594 on 7 January 2015. Downloaded from https://www.bmj.com/ on 3 March 2025 by guest. Protected by copyright, including for uses related to text and data mining, AI training, and similar technologies.
