# Supplementary material for: Prognostic ability of the sTarT back screening tool for disability and pain intensity outcomes in older adults with low back pain seeking chiropractic care: a multi-national external validation study
Source: Chiropr Man Therap. 2025 Jul 30;33:30. doi: 10.1186/s12998-025-00592-1 (PMC12312513; doi:10.1186/s12998-025-00592-1)
Supplement: Supplementary file 3 — Supplementary Material 3 [file 12998_2025_592_MOESM3_ESM.docx]

**Sensitivity analysis results in appendixes**

**Table 1s. Characteristics of the BACE-C patients at baseline among STarT Back risk groups in different countries (N=738)**

|  | SBT risk group (mean ± SD or n (%)) | | | P-value |
| --- | --- | --- | --- | --- |
|  | Low (n=130) * | Medium(n=67) * | High (n=18) * |  |
| **Netherlands (n=217)** |  |  |  |  |
| **Patient characteristics** |  |  |  |  |
| Age (years) | 66.9 ± 0.7 | 64.3 ± 0.9 | 66.9 ± 2.1 | 0.21 |
| Sex (female) | 64 (49.2) | 31 (46.3) | 4 (22.2) | 0.10 |
| Body mass index | 25.9 ± 0.4 | 26.8 ± 0.5 | 27.6 ± 1.5 | 0.13 |
| Educational level |  |  |  | 0.27 |
| low | 48 (36.9) | 30 (44.8) | 6 (33.3) |  |
| middle | 35 (26.9) | 12 (17.9) | 2 (11.1) |  |
| high | 47 (36.2) | 25 (37.3) | 10 (55.6) |  |
| Marital status |  |  |  |  |
| Married | 14 (10.8) | 14 (20.9) | 2 (11.1) | 0.31 |
| Living together | 5 (3.8) | 1 (1.5) | 1 (5.6) |  |
| Single | 111 (85.4) | 52 (77.6) | 15 (83.3) |  |
| Job (Employed) | 40 (30.8) | 29 (43.3) | 3 (16.7) | 0.02 |
| Heavy drinking risk  (high and severe risk) | 72 (55.4) | 38 (56.7) | 12 (66.7) | 0.58 |
| Smoke (Yes) | 8 (6.2) | 14 (20.9) | 3 (16.7) | 0.01 |
| **Pain back characteristics** |  |  |  |  |
| Back pain history (Yes) | 102 (78.5) | 56 (83.6) | 17 (94.4) | 0.23 |
| Chronicity (≥ 3 months) | 66 (50.8) | 34 (50.7) | 7 (38.9) | 0.79 |
| Radiating pain (Yes) | 64 (49.2) | 54 (80.6) | 11 (61.1) | < 0.001 |
| Back pain last week (NRS^a^) | 5.5 ± 1.9 | 6.6 ± 0.3 | 6.9 ± 0.5 | < 0.001 |
| Medication (Yes) | 53 (40.8) | 46 (68.7) | 11 (61.1) | < 0.001 |
| Comorbidity (SCQ^b^ sum score) | 4.5 ± 0.3 | 5.2 ± 0.6 | 6.2 ± 1.8 | 0.87 |
| Disability (RDQ^c^) | 4.5 ± 0.3 | 5.2 ± 0.6 | 6.2 ± 1.8 | < 0.001 |
| **Psychological characteristics** |  |  |  |  |
| EQ-5D-5L (VAS^d^) | 74.8 ± 1.4 | 63.7 ± 1.8 | 55.1 ± 4.0 | <0.001 |
| EQ-5D-5L (index^e^) | 0.8 ± 0.0 | 0.7 ± 0.0 | 0.4 ± 0.1 | <0.001 |
|  |  |  |  |  |
| **Sweden (n=301)** | **Low (n=190)** | **Medium(n=75)** | **High (n=10)** |  |
| **Patient characteristics** |  |  |  |  |
| Age (years) | 65.7 ± 0.5 | 63.2 ± 0.8 | 67.9 ± 2.4 | 0.04 |
| Sex (female) | 98 (51.6) | 46 (61.3) | 7 (70.0) | 0.27 |
| Body mass index | 26.3 ± 0.4 | 27.3 ±0.6 | 26.6 ± 1.4 | 0.10 |
| Educational level |  |  |  |  |
| low | 21 (11.1) | 6 (8.0) | 2 (20.0) | 0.62 |
| middle | 88 (46.3) | 41 (54.7) | 4 (40.0) |  |
| high | 81 (42.6) | 28 (37.3) | 4 (40.0) |  |
| Marital status |  |  |  |  |
| Married | 136 (71.6) | 58 (77.3) | 7 (70.0) | 0.59 |
| Living together | 9 (4.7) | 5 (6.7) | 0 (0) |  |
| Single | 44 (23.2) | 12 (16.0) | 3 (30.0) |  |
| Job (Employed) | 74 (38.9) | 39 (52.0) | 3 (30.0) | 0.02 |
| **Pain back characteristics** |  |  |  |  |
| Back pain history (Yes) | 147 (77.4) | 60 (80.0) | 6 (60.0) | 0.36 |
| Chronicity (≥ 3 months) | 86 (45.3) | 30 (40.0) | 3 (30.0) | 0.60 |
| Radiating pain (Yes) | 86 (45.3) | 14 (18.7) | 5 (50.0) | <0.001 |
| Back pain last week (NRS^a^) | 5.3 ± 0.2 | 7.3 ± 0.2 | 7.9 ± 0.6 | <0.001 |
| Medication (Yes) | 68 (35.8) | 36 (48.0) | 3 (30.0) | 0.32 |
| Comorbidity (SCQ^b^ sum score) | 1.6 ± 0.2 | 2.3 ± 0.3 | 1.0 ± 0.3 | 0.00 |
| Disability (RDQ^c^) | 7.3 ± 0.4 | 14.4 ± 0.5 | 17.1 ± 1.2 | <0.001 |
| **Psychological characteristics** |  |  |  |  |
| EQ-5D-3L (VAS^d^) | 76.8 ±1.2 | 61.8 ± 2.5 | 53.0 ± 8.0 | <0.001 |
| EQ-5D-3L (index^e^) | 0.9 ± 0.0 | 0.9 ± 0.0 | 0.8 ± 0.0 | <0.001 |
|  |  |  |  |  |
| **Australia (n=220)** | **Low (n=130)** | **Medium(n=59)** | **High (n=17)** |  |
| **Patient characteristics** |  |  |  |  |
| Age (years) | 67.3 ± 0.7 | 66.2 ± 1.1 | 69.6 ±2.3 | 0.28 |
| Sex (female) | 57 (43.8) | 35 (59.3) | 12 (70.6) | 0.14 |
| Body mass index | 27.1 ± 0.4 | 29.9 ± 0.9 | 26.7 ± 1.2 | 0.01 |
| Educational level |  |  |  |  |
| low | 35 (26.9) | 20 (33.9) | 9 (52.9) | 0.34 |
| middle | 18 (13.8) | 8 (13.6) | 0 (0) |  |
| high | 42 (32.3) | 19 (32.2) | 5 (29.4) |  |
| Vocational Education  (only for Australia) | 33 (25.4) | 11 (18.6) | 3 (17.6) |  |
| Marital status |  |  |  |  |
| Married | 34 (26.2) | 12 (20.3) | 4 (23.5) | 0.10 |
| Living together | 6 (4.6) | 11 18.6) | 3 (17.6) |  |
| Single | 80 (61.5) | 35 (59.3) | 9 (52.9) |  |
| Other (only for Australia) | 8 (6.2) | 1 (1.7) | 1 (5.9) |  |
| Job (Employed) | 20 (15.4) | 11 (18.6) | 3 (17.6) | 0.84 |
| Heavy drinking risk  (high and severe risk) | 22 (16.9) | 16 (27.1) | 2 (11.8) | 0.72 |
| Smoke (Yes) | 74 (56.9) | 35 (59.3) | 10 (58.8) | 0.76 |
| **Pain back characteristics** |  |  |  |  |
| Back pain history (Yes) | 108 (83.1) | 53 (89.8) | 15 (88.2) | 0.45 |
| Chronicity (≥ 3 months) | 50 (38.5) | 23 (39.0) | 8 (47.1) | 0.8 |
| Radiating pain (Yes) | 31 (23.8) | 23 (39.0) | 8 (47.0) | 0.05 |
| Back pain last week (NRS^a^) | 4.9 ± 0.2 | 6.5 ± 0.3 | 7.7 ± 0.6 | <0.001 |
| Medication (Yes) | 16 (12.3) | 17 (28.8) | 7 (41.2) | 0.002 |
| Comorbidity (SCQ^b^ sum score) | 1.5 ± 0.1 | 1.7 ± 0.2 | 2.1 ± 0.4 | 0.11 |
| Disability (RDQ^c^) | 4.3 ± 0.4 | 10.0 ± 0.5 | 15.4 ± 1.4 | <0.001 |
| **Psychological characteristics** |  |  |  |  |
| EQ-5D-3L (VAS^d^) | 76.7 ± 1.4 | 69.4 ± 2.2 | 60.6 ± 3.4 | <0.001 |
| EQ-5D-3L (index^e^) | 0.8 ± 0.1 | 0.6 ± 0.0 | 0.6 ± 0.0 | <0.001 |

a: the Numeric Rating Scale for pain; b: Self-Administered Comorbidities Questionnaire; c: 24-item Roland Morris Disability Questionnaire; d: Visual analogue scale; e: Health state index score of EQ5D5L and EQ5D3L; * Total number of patients in each risk group accounting missing values.

**Table 2s: Discrimination of the STarT Back risk subgroups for predicting *NO* improvement in disability and pain intensity in different sex subgroups**

|  | AUC (95%CI) | | Sensitivity | | Specificity | | PPV^f^ | | NPV^g^ | | LR+^h^ | | LR-^i^ | |
| --- | --- | --- | --- | --- | --- | --- | --- | --- | --- | --- | --- | --- | --- | --- |
|  | Male | Female | Male | Female | Male | Female | Male | Female | Male | Female | Male | Female | Male | Female |
| **SBT risk group cut-off: Low (n of male = 224; n of female = 219) VS Medium and High (n of male = 110; n of female = 135)** | | | | | | | | | | | | | | |
| Disability at 3 months | 0.523 (0.430, 0.615) | 0.546 (0.482, 0.610) | 0.361 | 0.443 | 0.684 | 0.649 | 0.350 | 0.405 | 0.695 | 0.684 | 1.143 | 1.261 | 0.934 | 0.859 |
| Disability at 6 months | 0.553 (0.475, 0.632) | 0.548 (0.479, 0.616) | 0.399 | 0.450 | 0.703 | 0.645 | 0.380 | 0.351 | 0.720 | 0.733 | 1.347 | 1.267 | 0.854 | 0.853 |
| Disability at 12 months | 0.547 (0.464, 0.630) | 0.530 (0.464, 0.596) | 0.399 | 0.426 | 0.695 | 0.635 | 0.323 | 0.334 | 0.761 | 0.720 | 1.309 | 1.168 | 0.864 | 0.903 |
| Pain at 3 months | 0.500 (0.428, 0.573) | 0.528 (0.453, 0.603) | 0.330 | 0.427 | 0.670 | 0.630 | 0.260 | 0.262 | 0.741 | 0.781 | 1.001 | 1.154 | 0.999 | 0.910 |
| Pain at 6 months | 0.554 (0.479, 0.629) | 0.539 (0.464, 0.614) | 0.410 | 0.444 | 0.699 | 0.634 | 0.331 | 0.258 | 0.765 | 0.799 | 1.361 | 1.213 | 0.844 | 0.877 |
| Pain at 12 months | 0.542 (0.464, 0.619) | 0.533 (0.458, 0.608) | 0.395 | 0.431 | 0.688 | 0.634 | 0.270 | 0.305 | 0.796 | 0.750 | 1.268 | 1.179 | 0.879 | 0.897 |
| **SBT risk group cut-off: Low and Medium (n of male = 312; n of female = 331) VS High (n of male = 22; n of female = 23)** | | | | | | | | | | | | | | |
| Disability at 3 months | 0.522 (0.451, 0.594) | 0.514 (0.450, 0.578) | 0.095 | 0.085 | 0.950 | 0.944 | 0.473 | 0.416 | 0.690 | 0.687 | 1.885 | 1.511 | 0.953 | 0.970 |
| Disability at 6 months | 0.518 (0.448, 0.589) | 0.522 (0.455, 0.590) | 0.090 | 0.097 | 0.947 | 0.947 | 0.436 | 0.456 | 0.696 | 0.698 | 1.684 | 1.834 | 0.962 | 0.953 |
| Disability at 12 months | 0.523 (0.448, 0.599) | 0.522 (0.457, 0.587) | 0.099 | 0.097 | 0.948 | 0.947 | 0.410 | 0.440 | 0.743 | 0.709 | 1.893 | 1.826 | 0.951 | 0.954 |
| Pain at 3 months | 0.505 (0.434, 0.575) | 0.507 (0.436, 0.577) | 0.072 | 0.077 | 0.938 | 0.937 | 0.288 | 0.273 | 0.743 | 0.767 | 1.154 | 1.216 | 0.990 | 0.985 |
| Pain at 6 months | 0.505 (0.433, 0.577) | 0.511 (0.436, 0.586) | 0.072 | 0.083 | 0.938 | 0.939 | 0.295 | 0.280 | 0.735 | 0.781 | 1.147 | 1.349 | 0.990 | 0.977 |
| Pain at 12 months | 0.515 (0.435, 0.596) | 0.519 (0.449, 0.589) | 0.088 | 0.093 | 0.942 | 0.994 | 0.307 | 0.385 | 0.780 | 0.737 | 1.514 | 1.664 | 0.968 | 0.961 |

f: positive predictive values**;** g: negative predictive values**;** h: Positive Likelihood Ratio; i: Negative Likelihood Ratio.

**Table 3s. Discrimination of the STarT Back risk subgroups for predicting *NO* improvement in disability and pain intensity in different countries subgroups**

|  | AUC (95%CI) | | | Sensitivity | | | Specificity | | | PPV^f^ | | | NPV^g^ | | | LR+^h^ | | | LR-^i^ | | |
| --- | --- | --- | --- | --- | --- | --- | --- | --- | --- | --- | --- | --- | --- | --- | --- | --- | --- | --- | --- | --- | --- |
|  | NL^j^ | SE^k^ | AU^l^ | NL | SE | AU | NL | SE | AU | NL | SE | AU | NL | SE | AU | NL | SE | AU | NL | SE | AU |
| **SBT risk group cut-off: Low (n of N = 130; n of S = 190; n of A = 130) VS Medium and High ((n of N = 85; n of S = 85; n of A = 76)** | | | | | | | | | | | | | | | | | | | | | |
| Disability at 3 months | 0.564 (0.451, 0.676) | 0.480 (0.403, 0.557) | 0.587 (0.504, 0.670) | 0.488 | 0.295 | 0.465 | 0.639 | 0.664 | 0.707 | 0.312 | 0.305 | 0.541 | 0.788 | 0.654 | 0.641 | 1.351 | 0.880 | 1.591 | 0.802 | 1.061 | 0.756 |
| Disability at 6 months | 0.615 (0.496, 0.733) | 0.483 (0.401, 0.564) | 0.584 (0.495, 0.672) | 0.570 | 0.297 | 0.464 | 0.659 | 0.668 | 0.704 | 0.327 | 0.262 | 0.529 | 0.841 | 0.706 | 0.647 | 1.672 | 0.895 | 1.566 | 0.652 | 1.052 | 0.762 |
| Disability at 12 months | 0.569 (0.448, 0.690) | 0.508 (0.424, 0.592) | 0.552 (0.470, 0.634) | 0.502 | 0.335 | 0.430 | 0.637 | 0.682 | 0.674 | 0.272 | 0.277 | 0.452 | 0.826 | 0.738 | 0.654 | 1.383 | 1.052 | 1.317 | 0.782 | 0.976 | 0.846 |
| Pain at 3 months | 0.539 (0.442, 0.637) | 0.503 (0.415, 0.590) | 0.488 (0.396, 0.580) | 0.450 | 0.326 | 0.349 | 0.629 | 0.679 | 0.627 | 0.308 | 0.200 | 0.283 | 0.757 | 0.804 | 0.696 | 1.212 | 1.016 | 0.936 | 0.875 | 0.993 | 1.038 |
| Pain at 6 months | 0.602 (0.500, 0.703) | 0.482 (0.388, 0.577) | 0.547 (0.443, 0.652) | 0.542 | 0.293 | 0.433 | 0.661 | 0.671 | 0.662 | 0.367 | 0.177 | 0.348 | 0.800 | 0.797 | 0.738 | 1.601 | 0.891 | 1.280 | 0.692 | 1.054 | 0.857 |
| Pain at 12 months | 0.572 (0.460, 0.7684) | 0.485 (0.390, 0.580) | 0.561 (0.470, 0.652) | 0.502 | 0.298 | 0.450 | 0.642 | 0.671 | 0.671 | 0.305 | 0.198 | 0.382 | 0.804 | 0.779 | 0.730 | 1.401 | 0.907 | 1.370 | 0.776 | 1.046 | 0.819 |
| **SBT risk group cut-off: Low and Medium (n of N = 197; n of S = 265; n of A = 189) VS High (n of N = 18; n of S = 10; n of A = 17)** | | | | | | | | | | | | | | | | | | | | | |
| Disability at 3 months | 0.542 (0.444, 0.641) | 0.511 (0.441, 0.581) | 0.508 (0.423, 0.592) | 0.147 | 0.054 | 0.092 | 0.939 | 0.968 | 0.924 | 0.447 | 0.459 | 0.475 | 0.766 | 0.672 | 0.579 | 2.388 | 1.694 | 1.208 | 0.909 | 0.977 | 0.983 |
| Disability at 6 months | 0.547 (0.443, 0.651) | 0.517 (0.433, 0.591) | 0.504 (0.422, 0.585) | 0.156 | 0.063 | 0.087 | 0.938 | 0.970 | 0.920 | 0.425 | 0.460 | 0.443 | 0.793 | 0.724 | 0.583 | 2.524 | 2.142 | 1.093 | 0.900 | 0.965 | 0.992 |
| Disability at 12 months | 0.560 (0.448, 0.672) | 0.526 (0.450, 0.602) | 0.490 (0.408, 0.572) | 0.177 | 0.077 | 0.071 | 0.943 | 0.975 | 0.910 | 0.458 | 0.529 | 0.331 | 0.809 | 0.744 | 0.610 | 3.077 | 3.071 | 0.787 | 0.874 | 0.946 | 1.021 |
| Pain at 3 months | 0.516 (0.426, 0.606) | 0.505 (0.421, 0.589) | 0.489 (0.401, 0.577) | 0.107 | 0.047 | 0.068 | 0.925 | 0.963 | 0.911 | 0.345 | 0.240 | 0.244 | 0.738 | 0.805 | 0.699 | 1.429 | 1.276 | 0.762 | 0.965 | 0.989 | 1.023 |
| Pain at 6 months | 0.528 (0.425, 0.632) | 0.503 (0.418, 0.589) | 0.485 (0.393, 0.577) | 0.125 | 0.044 | 0.062 | 0.932 | 0.962 | 0.909 | 0.404 | 0.222 | 0.222 | 0.747 | 0.806 | 0.700 | 1.844 | 1.168 | 0.676 | 0.939 | 0.993 | 1.033 |
| Pain at 12 months | 0.532 (0.428, 0.636) | 0.525 (0.441, 0.608) | 0.491 (0.401, 0.580) | 0.132 | 0.078 | 0.070 | 0.932 | 0.971 | 0.912 | 0.382 | 0.431 | 0.266 | 0.774 | 0.795 | 0.685 | 1.947 | 2.727 | 0.790 | 0.931 | 0.949 | 1.020 |

f: positive predictive values**;** g: negative predictive values**;** h: Positive Likelihood Ratio; i: Negative Likelihood Ratio; j: Netherlands; k: Sweden; l: Australia.

**Table 4s. Discrimination of the STarT Back risk subgroups for predicting *NO* improvement in disability and pain intensity in different duration subgroups**

|  | AUC (95%CI) | | Sensitivity | | Specificity | | PPV^f^ | | NPV^g^ | | LR+^h^ | | LR-^i^ | |
| --- | --- | --- | --- | --- | --- | --- | --- | --- | --- | --- | --- | --- | --- | --- |
|  | Acute | Chronic | Acute | Chronic | Acute | Chronic | Acute | Chronic | Acute | Chronic | Acute | Chronic | Acute | Chronic |
| **SBT risk group cut-off: Low (n of Acute = 228; n of Chronic = 202) VS Medium and High (n of Acute = 130; n of Chronic = 105)** | | | | | | | | | | | | | | |
| Disability at 3 months | 0.540 (0.461, 0.619) | 0.541 (0.470, 0.612) | 0.433 | 0.383 | 0.649 | 0.699 | 0.274 | 0.518 | 0.789 | 0.573 | 1.231 | 1.272 | 0.875 | 0.833 |
| Disability at 6 months | 0.562 (0.480, 0.644) | 0.548 (0.478, 0.617) | 0.468 | 0.395 | 0.657 | 0.700 | 0.279 | 0.478 | 0.813 | 0.625 | 1.362 | 1.318 | 0.811 | 0.864 |
| Disability at 12 months | 0.526 (0.454, 0.599) | 0.555 (0.486, 0.624) | 0.412 | 0.408 | 0.641 | 0.702 | 0.239 | 0.448 | 0.799 | 0.667 | 1.145 | 1.370 | 0.919 | 0.843 |
| Pain at 3 months | 0.493 (0.407, 0.579) | 0.534 (0.465, 0.603) | 0.358 | 0.382 | 0.628 | 0.686 | 0.149 | 0.407 | 0.843 | 0.663 | 0.962 | 1.215 | 1.023 | 0.901 |
| Pain at 6 months | 0.557 (0.474, 0.640) | 0.549 (0.480, 0.618) | 0.459 | 0.401 | 0.646 | 0.697 | 0.181 | 0.432 | 0.875 | 0.669 | 1.295 | 1.323 | 0.838 | 0.859 |
| Pain at 12 months | 0.535 (0.456, 0.614) | 0.548 (0.467, 0.628) | 0.428 | 0.401 | 0.642 | 0.694 | 0.202 | 0.405 | 0.842 | 0.690 | 1.196 | 1.310 | 0.891 | 0.863 |
| **SBT risk group cut-off: Low and Medium (n of Acute = 336; n of Chronic = 289) VS High (n of Acute = 22; n of Chronic = 18)** | | | | | | | | | | | | | | |
| Disability at 3 months | 0.512 (0.436, 0.588) | 0.528 (0.464, 0.591) | 0.089 | 0.089 | 0.935 | 0.967 | 0.297 | 0.692 | 0.770 | 0.556 | 1.372 | 2.653 | 0.974 | 0.943 |
| Disability at 6 months | 0.517 (0.446, 0.587) | 0.527 (0.460, 0.595) | 0.096 | 0.091 | 0.937 | 0.963 | 0.303 | 0.635 | 0.785 | 0.604 | 1.529 | 2.481 | 0.965 | 0.944 |
| Disability at 12 months | 0.524 (0.452, 0.596) | 0.524 (0.457, 0.590) | 0.108 | 0.088 | 0.940 | 0.959 | 0.332 | 0.562 | 0.794 | 0.640 | 1.809 | 2.156 | 0.949 | 0.951 |
| Pain at 3 months | 0.485 (0.406, 0.564) | 0.523 (0.456, 0.591) | 0.045 | 0.088 | 0.925 | 0.958 | 0.099 | 0.542 | 0.841 | 0.650 | 0.602 | 2.095 | 1.032 | 0.952 |
| Pain at 6 months | 0.495 (0.410, 0.580) | 0.520 (0.451, 0.588) | 0.061 | 0.083 | 0.928 | 0.956 | 0.128 | 0.521 | 0.853 | 0.645 | 0.858 | 1.878 | 1.011 | 0.959 |
| Pain at 12 months | 0.509 (0.432, 0.586) | 0.526 (0.454, 0.597) | 0.085 | 0.093 | 0.933 | 0.959 | 0.212 | 0.545 | 0.829 | 0.670 | 1.269 | 2.279 | 0.981 | 0.946 |

f: positive predictive values**;** g: negative predictive values**;** h: Positive Likelihood Ratio; i: Negative Likelihood Ratio

**Table 5s. Predictive ability of the STarT Back risk subgroups for predicting disability and pain intensity linear model for continuous outcomes**

|  | R square | RMSE |
| --- | --- | --- |
| Disability at 3 months | 0.183 | 4.557 |
| Disability at 6 months | 0.163 | 4.693 |
| Disability at 12 months | 0.143 | 4.697 |
| Pain at 3 months | 0.057 | 2.171 |
| Pain at 6 months | 0.079 | 2.247 |
| Pain at 12 months | 0.083 | 2.206 |

**Table 6s. Predictive ability of the STarT Back risk subgroups for predicting disability and pain intensity linear model for continuous outcomes in different sex subgroups**

|  | R square | | RMSE | |
| --- | --- | --- | --- | --- |
|  | Male | Female | Male | Female |
| Disability at 3 months | 0.133 | 0.233 | 4.483 | 4.584 |
| Disability at 6 months | 0.119 | 0.210 | 4.595 | 4.764 |
| Disability at 12 months | 0.130 | 0.160 | 4.374 | 4.947 |
| Pain at 3 months | 0.033 | 0.090 | 2.207 | 2.145 |
| Pain at 6 months | 0.058 | 0.109 | 2.363 | 2.138 |
| Pain at 12 months | 0.069 | 0.100 | 2.175 | 2.234 |

**Table 7s. Predictive ability of the STarT Back risk subgroups for predicting disability and pain intensity linear model for continuous outcomes in different countries subgroups.**

|  | R square | | | RMSE | | |
| --- | --- | --- | --- | --- | --- | --- |
|  | NL^j^ | SE^k^ | AU^l^ | NL | SE | AU |
| Disability at 3 months | 0.171 | 0.139 | 0.309 | 4.503 | 4.793 | 4.148 |
| Disability at 6 months | 0.156 | 0.122 | 0.263 | 4.460 | 4.723 | 4.739 |
| Disability at 12 months | 0.118 | 0.141 | 0.203 | 4.692 | 4.722 | 4.598 |
| Pain at 3 months | 0.081 | 0.026 | 0.079 | 2.245 | 2.099 | 2.155 |
| Pain at 6 months | 0.107 | 0.043 | 0.107 | 2.263 | 2.147 | 2.331 |
| Pain at 12 months | 0.077 | 0.086 | 0.103 | 2.260 | 2.090 | 2.272 |

j: Netherlands; k: Sweden; l: Australia

**Table 8s. Predictive ability of the STarT Back risk subgroups for predicting disability and pain intensity linear model for continuous outcomes in different duration subgroups.**

|  | R square | | RMSE | |
| --- | --- | --- | --- | --- |
|  | Acute | Chronic | Acute | Chronic |
| Disability at 3 months | 0.164 | 0.248 | 4.129 | 4.674 |
| Disability at 6 months | 0.161 | 0.200 | 4.321 | 4.857 |
| Disability at 12 months | 0.117 | 0.204 | 4.281 | 4.895 |
| Pain at 3 months | 0.036 | 0.134 | 1.842 | 2.251 |
| Pain at 6 months | 0.085 | 0.116 | 1.910 | 2.361 |
| Pain at 12 months | 0.066 | 0.127 | 2.036 | 2.269 |

**Table 9s: Discrimination of the STarT Back risk subgroups for predicting *NO* improvement disability based on the Hill’s definition.**

|  | AUC (95%CI) | Sensitivity | Specificity | PPV^f^ | NPVg | LR+^h^ | LR- ^i^ |
| --- | --- | --- | --- | --- | --- | --- | --- |
| **SBT risk group cut-off: Low (n=450) VS Medium and High (n=246)** | | | | | | | |
| Disability at 3 months | 0.698 (0.648-0.749) | 0.645 | 0.752 | 0.492 | 0.850 | 2.597 | 0.473 |
| Disability at 6 months | 0.638 (0.634-0.732) | 0.627 | 0.740 | 0.461 | 0.848 | 2.409 | 0.504 |
| Disability at 12 months | 0.670 (0.619-0.720) | 0.611 | 0.728 | 0.425 | 0.850 | 2.244 | 0.0.534 |
| **SBT risk group cut-off: Low and Medium (n=651) VS High (n=45)** | | | | | | | |
| Disability at 3 months | 0.555 (0.500-0.610) | 0.145 | 0.965 | 0.608 | 0.751 | 4.114 | 0.886 |
| Disability at 6 months | 0.552 (0.500-0.604) | 0.142 | 0.962 | 0.571 | 0.760 | 3.751 | 0.892 |
| Disability at 12 months | 0.562 (0.504-0.621) | 0.159 | 0.966 | 0.608 | 0.777 | 4.671 | 0.871 |

f: positive predictive values**;** g: negative predictive values**;** h: Positive Likelihood Ratio; i: Negative Likelihood Ratio

**Table 10s: Discrimination of the STarT Back risk subgroups for predicting *NO* improvement disability based on the Hill’s definition in different sex subgroups.**

|  | AUC (95%CI) | | Sensitivity | | Specificity | | PPV^f^ | | NPV^g^ | | LR+^h^ | | LR-^i^ | |
| --- | --- | --- | --- | --- | --- | --- | --- | --- | --- | --- | --- | --- | --- | --- |
|  | Male | Female | Male | Female | Male | Female | Male | Female | Male | Female | Male | Female | Male | Female |
| **SBT risk group cut-off: Low (n of male = 224; n of female = 219) VS Medium and High (n of male = 110; n of female = 135)** | | | | | | | | | | | | | | |
| Disability at 3 months | 0.665 (0.577, 0.753) | 0.726 (0.666, 0.785) | 0.586 | 0.693 | 0.745 | 0.759 | 0.403 | 0.568 | 0.860 | 0.844 | 2.296 | 2.871 | 0.556 | 0.405 |
| Disability at 6 months | 0.660 (0.580, 0.741) | 0.702 (0.634, 0.770) | 0.572 | 0.675 | 0.748 | 0.730 | 0.423 | 0.493 | 0.844 | 0.852 | 2.273 | 2.497 | 0.572 | 0.446 |
| Disability at 12 months | 0.680 (0.583, 0.777) | 0.662 (0.594, 0.729) | 0.613 | 0.616 | 0.746 | 0.707 | 0.395 | 0.450 | 0.878 | 0.826 | 2.415 | 2.105 | 0.519 | 0.543 |
| **SBT risk group cut-off: Low and Medium (n of male = 312; n of female = 331) VS High (n of male = 22; n of female = 23)** | | | | | | | | | | | | | | |
| Disability at 3 months | 0.559 (0.476, 0.643) | 0.553 (0.484, 0.622) | 0.156 | 0.138 | 0.962 | 0.967 | 0.553 | 0.658 | 0.795 | 0.710 | 4.154 | 4.169 | 0.877 | 0.891 |
| Disability at 6 months | 0.543 (0.465, 0.620) | 0.561 (0.492, 0.630) | 0.129 | 0.153 | 0.956 | 0.968 | 0.488 | 0.652 | 0.773 | 0.746 | 2.936 | 4.764 | 0.911 | 0.875 |
| Disability at 12 months | 0.564 (0.471, 0.658) | 0.562 (0.491, 0.633) | 0.166 | 0.155 | 0.963 | 0.968 | 0.553 | 0.658 | 0.811 | 0.746 | 4.468 | 4.911 | 0.866 | 0.872 |

f: positive predictive values**;** g: negative predictive values**;** h: Positive Likelihood Ratio; i: Negative Likelihood Ratio

**Table 11s: Discrimination of the STarT Back risk subgroups for predicting *NO* improvement disability based on the Hill’s definition in different country subgroups.**

|  | AUC (95%CI) | | | Sensitivity | | | Specificity | | | PPV^f^ | | | NPV^g^ | | | LR+^h^ | | | LR-^i^ | | |
| --- | --- | --- | --- | --- | --- | --- | --- | --- | --- | --- | --- | --- | --- | --- | --- | --- | --- | --- | --- | --- | --- |
|  | NL^j^ | SE^k^ | AU^l^ | NL | SE | AU | NL | SE | AU | NL | SE | AU | NL | SE | AU | NL | SE | AU | NL | SE | AU |
| **SBT risk group cut-off: Low (n of N = 130; n of S = 190; n of A = 130) VS Medium and High ((n of N = 85; n of S = 85; n of A = 76)** | | | | | | | | | | | | | | | | | | | | | |
| Disability at 3 months | 0.717 (0.614, 0.821) | 0.641 (0.564, 0.718) | 0.768 (0.693, 0.843) | 0.725 | 0.524 | 0.739 | 0.703 | 0.759 | 0.797 | 0.406 | 0.466 | 0.613 | 0.901 | 0.799 | 0.875 | 2.438 | 2.176 | 3.665 | 0.391 | 0.627 | 0.327 |
| Disability at 6 months | 0.717 (0.6214, 0.813) | 0.629 (0.549, 0.709) | 0.730 (0.648, 0.812) | 0.727 | 0.512 | 0.694 | 0.707 | 0.746 | 0.766 | 0.425 | 0.421 | 0.546 | 0.897 | 0.809 | 0.861 | 2.481 | 2.014 | 2.970 | 0.386 | 0.655 | 0.399 |
| Disability at 12 months | 0.673 (0.556, 0.790) | 0.641 (0.552, 0.729) | 0.709 (0.624, 0.795 | 0. 666 | 0.532 | 0.659 | 0.680 | 0.749 | 0.750 | 0.355 | 0.417 | 0.505 | 0.886 | 0.826 | 0.851 | 2.082 | 2.117 | 2.639 | 0.491 | 0.625 | 0.454 |
| **SBT risk group cut-off: Low and Medium (n of N = 197; n of S = 265; n of A = 189) VS High (n of N = 18; n of S = 10; n of A = 17)** | | | | | | | | | | | | | | | | | | | | | |
| Disability at 3 months | 0.568 (0.455, 0.682) | 0.534 (0.459, 0.609) | 0.574 (0.478, 0.669) | 0.189 | 0.088 | 0.186 | 0.947 | 0.981 | 0.962 | 0.504 | 0.647 | 0.689 | 0.806 | 0.728 | 0.731 | 3.560 | 4.536 | 4.936 | 0.856 | 0.930 | 0.846 |
| Disability at 6 months | 0.540 (0.435, 0.645) | 0.540 (0.462, 0.618) | 0.579 (0.487, 0.671) | 0.145 | 0.098 | 0.195 | 0.935 | 0.982 | 0.963 | 0.403 | 0.665 | 0.684 | 0.786 | 0.751 | 0.747 | 2.241 | 5.385 | 5.276 | 0.914 | 0.919 | 0.836 |
| Disability at 12 months | 0.572 (0.454, 0.691) | 0.546 (0.446, 0.626) | 0.576 (0.485, 0.667) | 0.197 | 0.109 | 0.192 | 0.948 | 0.984 | 0.960 | 0.506 | 0.697 | 0.649 | 0.818 | 0.766 | 0.754 | 3.770 | 6.787 | 4.743 | 0.847 | 0.906 | 0.842 |

f: positive predictive values**;** g: negative predictive values**;** h: Positive Likelihood Ratio; i: Negative Likelihood Ratio; j: Netherlands; k: Sweden; l: Australia

**Table 12s: Discrimination of the STarT Back risk subgroups for predicting *NO* improvement in disability and pain intensity on the Hill’s definition in different duration subgroups.**

|  | AUC (95%CI) | | Sensitivity | | Specificity | | PPV^f^ | | NPV^g^ | | LR+^h^ | | LR-^i^ | |
| --- | --- | --- | --- | --- | --- | --- | --- | --- | --- | --- | --- | --- | --- | --- |
|  | Acute | Chronic | Acute | Chronic | Acute | Chronic | Acute | Chronic | Acute | Chronic | Acute | Chronic | Acute | Chronic |
| **SBT risk group cut-off: Low (n of Acute = 228; n of Chronic = 202 ) VS Medium and High (n of Acute = 130; n of Chronic = 105)** | | | | | | | | | | | | | | |
| Disability at 3 months | 0.702 (0.630, 0.773) | 0.711 (0.644, 0.778) | 0.692 | 0.612 | 0.712 | 0.810 | 0.383 | 0.637 | 0.900 | 0.793 | 2.406 | 3.228 | 0.432 | 0.479 |
| Disability at 6 months | 0.689 (0.619, 0.760) | 0.695 (0.627, 0.763) | 0.667 | 0.594 | 0.702 | 0.796 | 0.350 | 0.607 | 0.902 | 0.787 | 2.270 | 2.917 | 0.461 | 0.510 |
| Disability at 12 months | 0.655 (0.581, 0.728) | 0.696 (0.627, 0.765) | 0.622 | 0.604 | 0.687 | 0.787 | 0.313 | 0.573 | 0.888 | 0.807 | 1.990 | 2.834 | 0.549 | 0.503 |
| **SBT risk group cut-off: Low and Medium (n of Acute = 336; n of Chronic = 289) VS High (n of Acute = 22; n of Chronic = 18)** | | | | | | | | | | | | | | |
| Disability at 3 months | 0.559 (0.484, 0.634) | 0.556 (0.480, 0.632) | 0.164 | 0.132 | 0.954 | 0.981 | 0.479 | 0.793 | 0.816 | 0.675 | 3.550 | 6.784 | 0.876 | 0.886 |
| Disability at 6 months | 0.555 (0.478, 0.632) | 0.554 (0.484, 0.624) | 0.160 | 0.130 | 0.951 | 0.979 | 0.437 | 0.766 | 0.827 | 0.680 | 3.269 | 6.113 | 0.883 | 0.889 |
| Disability at 12 months | 0.578 (0.493, 0.662) | 0.578 (0.493-0.622) | 0.197 | 0.132 | 0.959 | 0.976 | 0.523 | 0.731 | 0.839 | 0.703 | 4.749 | 5.563 | 0.838 | 0.889 |

f: positive predictive values**;** g: negative predictive values**;** h: Positive Likelihood Ratio; i: Negative Likelihood Ratio

**Table 13s. Discrimination of the STarT Back sum score for predicting *NO* improvement in disability and pain intensity in different sex subgroups**

|  | AUC (95%CI) | | Sensitivity | | Specificity | | PPV^f^ | | NPV^g^ | | LR+^h^ | | LR-^i^ | |
| --- | --- | --- | --- | --- | --- | --- | --- | --- | --- | --- | --- | --- | --- | --- |
|  | Male | Female | Male | Female | Male | Female | Male | Female | Male | Female | Male | Female | Male | Female |
| Disability at 3 months | 0.522 (0.415, 0.629) | 0.530 (0.463, 0.596) | 0.364 | 0.436 | 0.690 | 0.648 | 0.357 | 0.400 | 0.689 | 0.681 | 1.176 | 1.238 | 0.921 | 0.871 |
| Disability at 6 months | 0.551 (0.450, 0.651) | 0.546 (0.471, 0.621) | 0.403 | 0.450 | 0.708 | 0.648 | 0.385 | 0.353 | 0.723 | 0.734 | 1.378 | 1.276 | 0.844 | 0.850 |
| Disability at 12 months | 0.551 (0.465, 0.636) | 0.532 (0.459, 0.605) | 0.399 | 0.421 | 0.699 | 0.636 | 0.326 | 0.332 | 0.762 | 0.718 | 1.325 | 1.154 | 0.860 | 0.912 |
| Pain at 3 months | 0.504 (0.421, 0.587) | 0.505 (0.426, 0.585) | 0.326 | 0. 418 | 0.672 | 0.631 | 0.258 | 0.259 | 0.740 | 0.779 | 0.992 | 1.132 | 1.004 | 0.923 |
| Pain at 6 months | 0.542 (0.456, 0.627) | 0.497 (0.402, 0.592) | 0.414 | 0.439 | 0.704 | 0.635 | 0.337 | 0.257 | 0.768 | 0.798 | 1.400 | 1.204 | 0.832 | 0.883 |
| Pain at 12 months | 0.537 (0.449, 0.625) | 0.497 (0.406, 0.589) | 0.395 | 0.432 | 0.692 | 0.638 | 0.272 | 0.308 | 0.797 | 0.751 | 1.280 | 1.193 | 0.875 | 0.890 |

f: positive predictive values**;** g: negative predictive values**;** h: Positive Likelihood Ratio; i: Negative Likelihood Ratio

**Table 14s. Discrimination of the STarT Back sum score for predicting *NO* improvement in disability and pain intensity in different countries subgroups**

|  | AUC (95%CI) | | | Sensitivity | | | Specificity | | | PPV^f^ | | | NPV^g^ | | | LR+^h^ | | | LR-^i^ | | |
| --- | --- | --- | --- | --- | --- | --- | --- | --- | --- | --- | --- | --- | --- | --- | --- | --- | --- | --- | --- | --- | --- |
|  | NL^j^ | SE^k^ | AU^l^ | NL | SE | AU | NL | SE | AU | NL | SE | AU | NL | SE | AU | NL | SE | AU | NL | SE | AU |
| Disability at 3 months | 0.581 (0.458, 0.704) | 0.461 (0.385, 0.536) | 0.572 (0.477, 0.667) | 0.447 | 0.276 | 0.472 | 0.624 | 0.674 | 0.708 | 0.297 | 0.297 | 0.545 | 0.761 | 0.651 | 0.644 | 1.192 | 0.844 | 1.614 | 0.886 | 1.075 | 0.746 |
| Disability at 6 months | 0.615 (0.478, 0.752) | 0.461 (0.374, 0.549) | 0.594 (0.500, 0.688) | 0.520 | 0.273 | 0.472 | 0.644 | 0.677 | 0.705 | 0.311 | 0.250 | 0.534 | 0.813 | 0.702 | 0.651 | 1.468 | 0.844 | 1.600 | 0.747 | 1.075 | 0.749 |
| Disability at 12 months | 0.581 (0.440, 0.723) | 0.498 (0.411, 0.585) | 0.562 (0.474, 0.651) | 0.493 | 0.306 | 0.441 | 0.636 | 0.690 | 0.675 | 0.292 | 0.264 | 0.459 | 0.805 | 0.732 | 0.659 | 1.358 | 1.987 | 1.356 | 0.797 | 1.006 | 0.828 |
| Pain at 3 months | 0.525 (0.417, 0.633) | 0.468 (0.380, 0.555) | 0.493 (0.395, 0.591) | 0.458 | 0.301 | 0.349 | 0.630 | 0.689 | 0.623 | 0.320 | 0.192 | 0.281 | 0.754 | 0.800 | 0.695 | 1.238 | 0.967 | 0.927 | 0.861 | 1.015 | 1.044 |
| Pain at 6 months | 0.584 (0.471, 0.697) | 0.412 (0.310, 0.513) | 0.530 (0.412, 0.648) | 0.534 | 0.268 | 0.440 | 0.655 | 0.681 | 0.662 | 0.355 | 0.169 | 0.351 | 0.798 | 0.794 | 0.740 | 1.549 | 0.840 | 1.300 | 0.711 | 1.075 | 0.846 |
| Pain at 12 months | 0.545 (0.425, 0.666) | 0.430 (0.310, 0.549) | 0.559 (0.454, 0.664) | 0.486 | 0.278 | 0.458 | 0.638 | 0.683 | 0.673 | 0.315 | 0.193 | 0.387 | 0.784 | 0.777 | 0.734 | 1.345 | 0.878 | 1.400 | 0.808 | 1.057 | 0.806 |

f: positive predictive values**;** g: negative predictive values**;** h: Positive Likelihood Ratio; i: Negative Likelihood Ratio; j: Netherlands; k: Sweden; l: Australia

**Table 15s. Discrimination of the STarT Back sum score for predicting *NO* improvement in disability and pain intensity in different duration subgroups**

|  | AUC (95%CI) | | Sensitivity | | Specificity | | PPV^f^ | | NPV^g^ | | LR+^h^ | | LR-^i^ | |
| --- | --- | --- | --- | --- | --- | --- | --- | --- | --- | --- | --- | --- | --- | --- |
|  | Acute | Chronic | Acute | Chronic | Acute | Chronic | Acute | Chronic | Acute | Chronic | Acute | Chronic | Acute | Chronic |
| Disability at 3 months | 0.509 (0.415, 0.5603) | 0.556 (0.482, 0.629) | 0.421 | 0.386 | 0.653 | 0.698 | 0.271 | 0.522 | 0.786 | 0.571 | 1.212 | 1.275 | 0.887 | 0.881 |
| Disability at 6 months | 0.554 (0.464, 0.644) | 0.556 (0.478, 0.635) | 0.459 | 0.400 | 0.662 | 0.701 | 0.278 | 0.481 | 0.812 | 0.627 | 1.358 | 1.335 | 0.817 | 0.857 |
| Disability at 12 months | 0.525 (0.452, 0.598) | 0.563 (0.494, 0.632) | 0.395 | 0.414 | 0.644 | 0.702 | 0.233 | 0.451 | 0.795 | 0.670 | 1.108 | 1.389 | 0.940 | 0.835 |
| Pain at 3 months | 0.467 (0.377, 0.558) | 0.535 (0.459, 0.611) | 0.353 | 0.375 | 0.634 | 0.679 | 0.150 | 0.397 | 0.843 | 0.658 | 0.964 | 1.168 | 1.021 | 0.921 |
| Pain at 6 months | 0.522 (0.429, 0.614) | 0.525 (0.448, 0.603) | 0.459 | 0.404 | 0.652 | 0.695 | 0.183 | 0.432 | 0.876 | 0.670 | 1.317 | 1.326 | 0.831 | 0.857 |
| Pain at 12 months | 0.512 (0.424, 0.601) | 0.525 (0.443, 0.607) | 0.415 | 0.409 | 0.647 | 0.694 | 0.199 | 0.410 | 0.840 | 0.693 | 1.175 | 1.338 | 0.904 | 0.851 |

f: positive predictive values**;** g: negative predictive values**;** h: Positive Likelihood Ratio; i: Negative Likelihood Ratio
